# The genome sequence of the springtail, *Dicyrtomina minuta *(O.Fabricius, 1783)

**DOI:** 10.12688/wellcomeopenres.22765.1

**Published:** 2024-07-29

**Authors:** Kamil S. Jaron, Clément Schneider, Christina N. Hodson, University of Oxford and Wytham Woods Genome Acquisition Lab, Darwin Tree of Life Barcoding collective, Wellcome Sanger Institute Tree of Life Management, Samples and Laboratory team, Wellcome Sanger Institute Scientific Operations: Sequencing Operations, Wellcome Sanger Institute Tree of Life Core Informatics team, Tree of Life Core Informatics collective, Darwin Tree of Life Consortium

**Affiliations:** 1 Tree of Life, Wellcome Sanger Institute, Hinxton, England, UK; 2 Senckenberg Museum of Natural History, Görlitz, Germany; 3 University of British Columbia, Biodiversity Research Centre, Canada

**Keywords:** Dicyrtomina minuta, springtail, genome sequence, chromosomal, Symphypleona

## Abstract

We present a genome assembly from an individual female *Dicyrtomina minuta* (springtail; Arthropoda; Collembola; Symphypleona; Dicyrtomidae). The genome sequence is 582.0 megabases in span. Most of the assembly is scaffolded into 5 chromosomal pseudomolecules, including the X _1_ and X _2_ sex chromosomes. The mitochondrial genome has also been assembled and is 15.59 kilobases in length.

## Species taxonomy

Eukaryota; Opisthokonta; Metazoa; Eumetazoa; Bilateria; Protostomia; Ecdysozoa; Panarthropoda; Arthropoda; Mandibulata; Pancrustacea; Hexapoda; Collembola; Symphypleona; Dicyrtomidae; Dicyrtominae; *Dicyrtomina*; *Dicyrtomina minuta* (O.Fabricius, 1783) (NCBI:txid1387116).

## Background

Springtails are one of the most abundant groups of soil animals, found in all sorts of biomes and habitats worldwide ( Hopkin, 1997). Most of the springtails are truly miniature creatures, often smaller than a millimetre, but there are exceptions. *Dicyrtomina minuta* is one of the larger globular springtails and is up to 3mm long, which is a paradox given its species name (since *minuta* means small). These springtails can be found in moist leaf litter in Europe and North America during autumn and winter, alongside other species of the same family Dicyrtomidae ( GBIF Secretariat, 2024). The fourth antenatal segment of Dicyrtomidae is very short, which is a distinct feature that can be used for quick identification to family level. Another remarkable trait of the family is the secretion of numerous rods of wax, secreted by special chaetae ( Massoud & Vannier, 1965). The rods are sheddable, and their function remains unclear, presumably involved in the sensorial system. Three species of the *Dicyrtomina* genus, *D. minuta, D. ornata* and *D. saudersi* differ only in colour pattern variations and therefore were considered by some authors morphs of the same species ( Richards, 1968).

In general, karyotypes of globular springtails (Symphypleona) are thought to be conserved, with 2n=12 chromosomes in females and 2n=10 in males ( Dallai *et al.*, 2004), which was also observed in *D. ornate* ( Dallai *et al.*, 1999). The only exception is a more distantly related member Dicyrtomidae, *Ptenothrix italica*, with 2n=14 and 2n=12 in males ( Dallai *et al.*, 1999). However, the karyotype of *D. minuta* was prior to this study unknown. Based on the overall conservation of the double X chromosome system (X _1_X _2_00) and aberrant spermatogenesis in Symphypleona ( Dallai *et al.*, 1999), it is expected that this species is reproducing via Paternal Genome Elimination ( Jaron *et al.*, 2022), a reproductive mode where males eliminate the paternal genome, and pass only the maternal one to the next generation. However, this hypothesis has not been directly tested in this species yet.

The genome of a springtail, *Dicyrtomina minuta*, was sequenced as part of the Darwin Tree of Life Project, a collaborative effort to sequence all named eukaryotic species in the Atlantic Archipelago of Britain and Ireland. Here we present a chromosomally complete genome sequence for *Dicyrtomina minuta*, based on a female specimen from Wytham Woods, Oxfordshire, UK.

## Genome sequence report

The genome was sequenced from an adult female *Dicyrtomina minuta* collected from Wytham Woods, Berkshire, UK (51.78, –1.34). A total of 39-fold coverage in Pacific Biosciences single-molecule HiFi long reads was generated. Primary assembly contigs were scaffolded with chromosome conformation Hi-C data. Manual assembly curation corrected 68 missing joins or mis-joins and removed 8 haplotypic duplications, reducing the assembly length by 0.70% and the scaffold number by 6.36%, and increasing the scaffold N50 by 2.24%.

**Figure 1.**
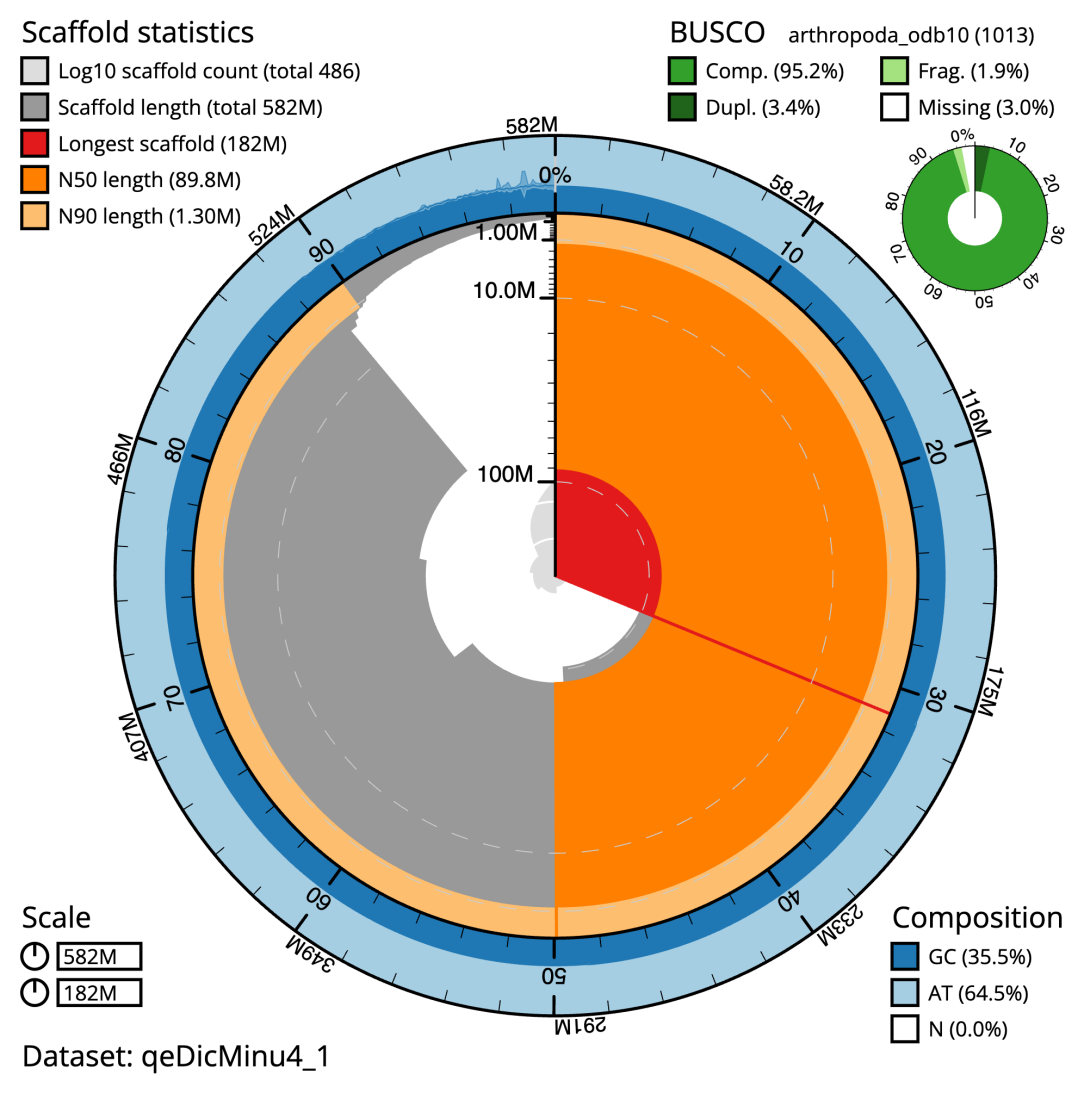
Genome assembly of *Dicyrtomina minuta*, qeDicMinu4.1: metrics. The BlobToolKit snail plot shows N50 metrics and BUSCO gene completeness. The main plot is divided into 1,000 size-ordered bins around the circumference with each bin representing 0.1% of the 581,995,013 bp assembly. The distribution of scaffold lengths is shown in dark grey with the plot radius scaled to the longest scaffold present in the assembly (181,988,040 bp, shown in red). Orange and pale-orange arcs show the N50 and N90 scaffold lengths (89,754,956 and 1,302,000 bp), respectively. The pale grey spiral shows the cumulative scaffold count on a log scale with white scale lines showing successive orders of magnitude. The blue and pale-blue area around the outside of the plot shows the distribution of GC, AT and N percentages in the same bins as the inner plot. A summary of complete, fragmented, duplicated and missing BUSCO genes in the arthropoda_odb10 set is shown in the top right. An interactive version of this figure is available at https://blobtoolkit.genomehubs.org/view/Dicyrtomina%20minuta/dataset/qeDicMinu4_1/snail.

**Figure 2.**
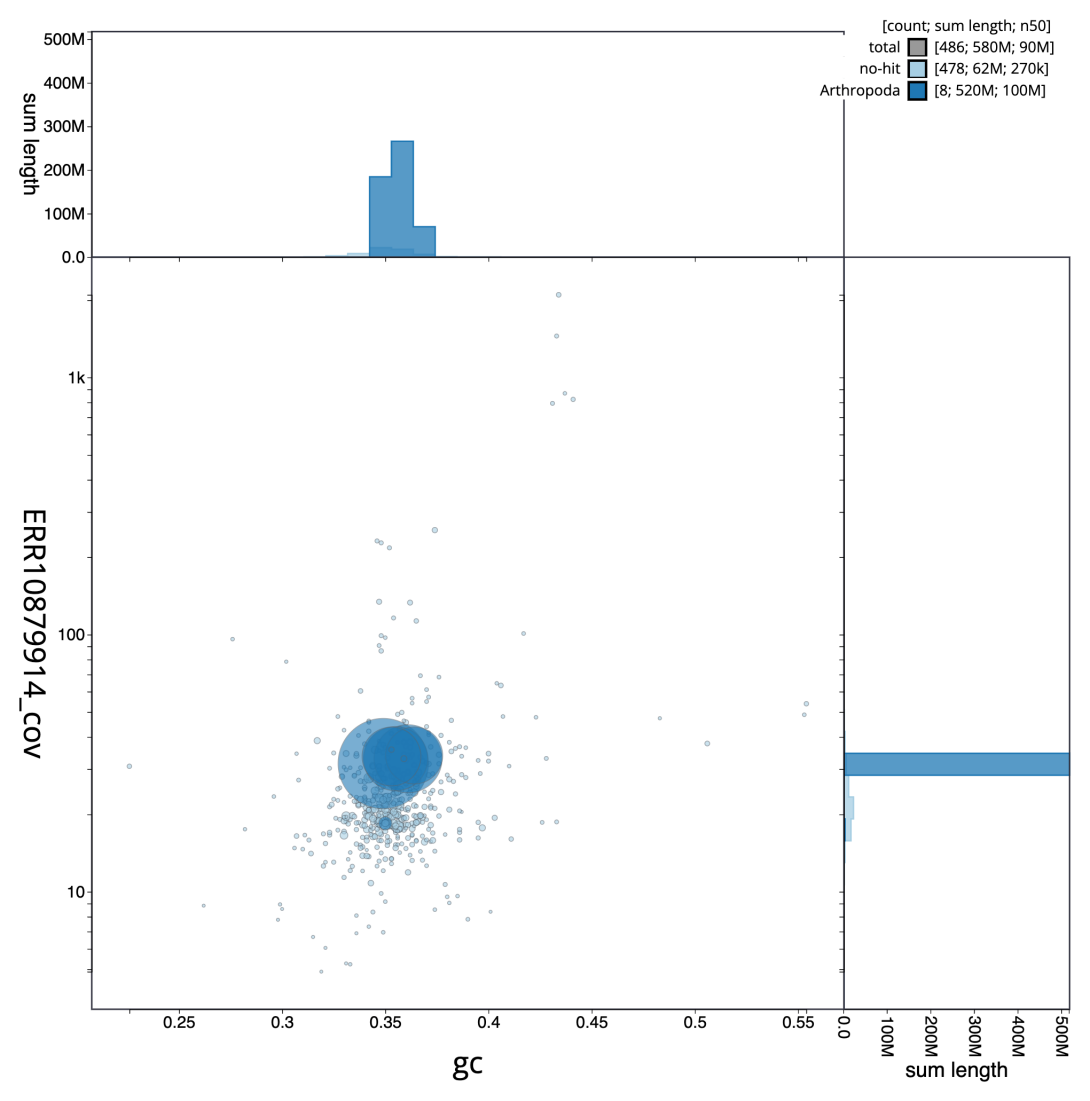
Genome assembly of *Dicyrtomina minuta*, qeDicMinu4.1: BlobToolKit GC-coverage plot. Sequences are coloured by phylum. Circles are sized in proportion to sequence length. Histograms show the distribution of sequence length sum along each axis. An interactive version of this figure is available at https://blobtoolkit.genomehubs.org/view/Dicyrtomina%20minuta/dataset/qeDicMinu4_1/blob.

**Figure 3.**
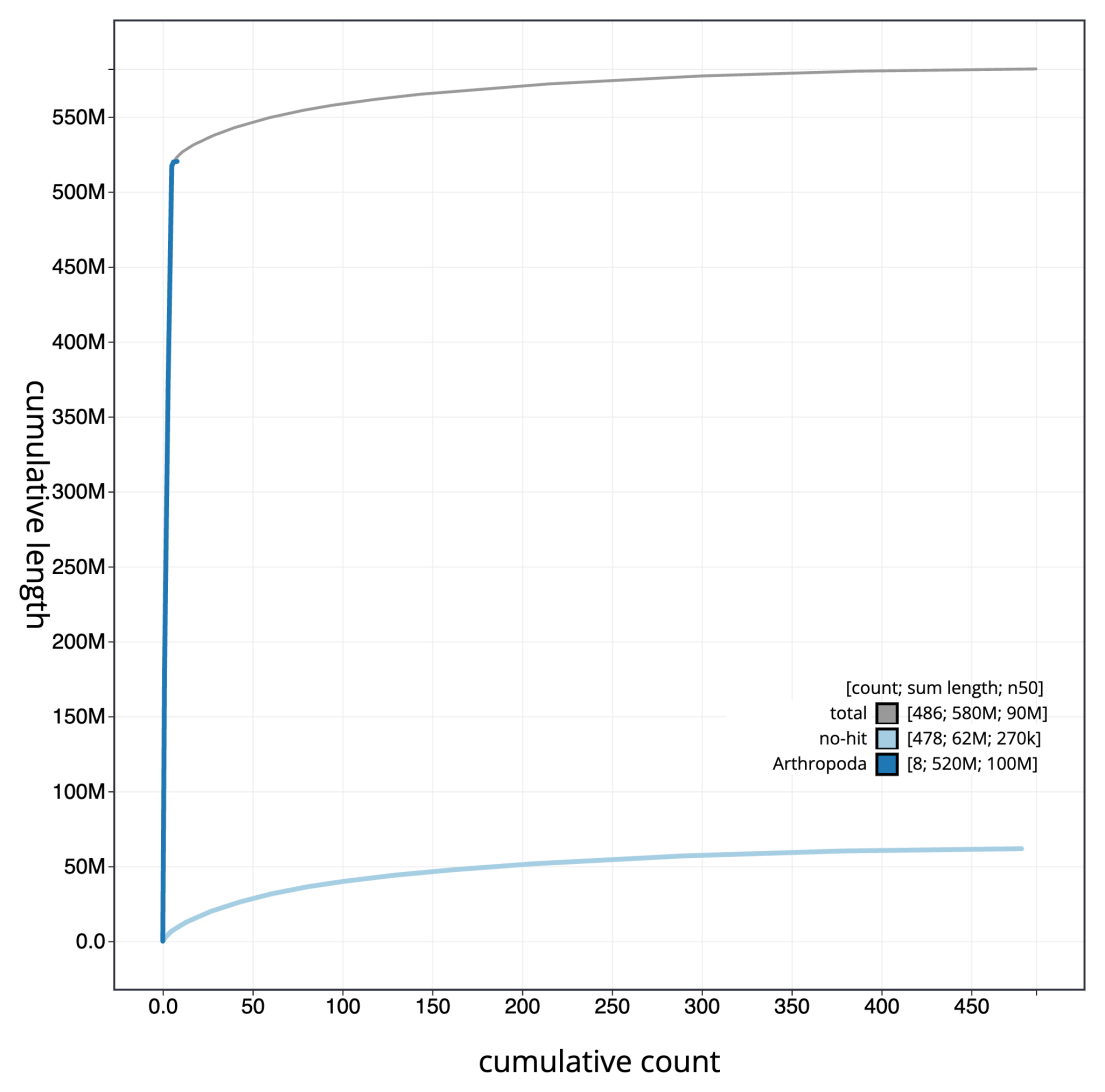
Genome assembly of *Dicyrtomina minuta* qeDicMinu4.1: BlobToolKit cumulative sequence plot. The grey line shows cumulative length for all sequences. Coloured lines show cumulative lengths of sequences assigned to each phylum using the buscogenes taxrule. An interactive version of this figure is available at https://blobtoolkit.genomehubs.org/view/Dicyrtomina%20minuta/dataset/qeDicMinu4_1/cumulative.

**Figure 4.**
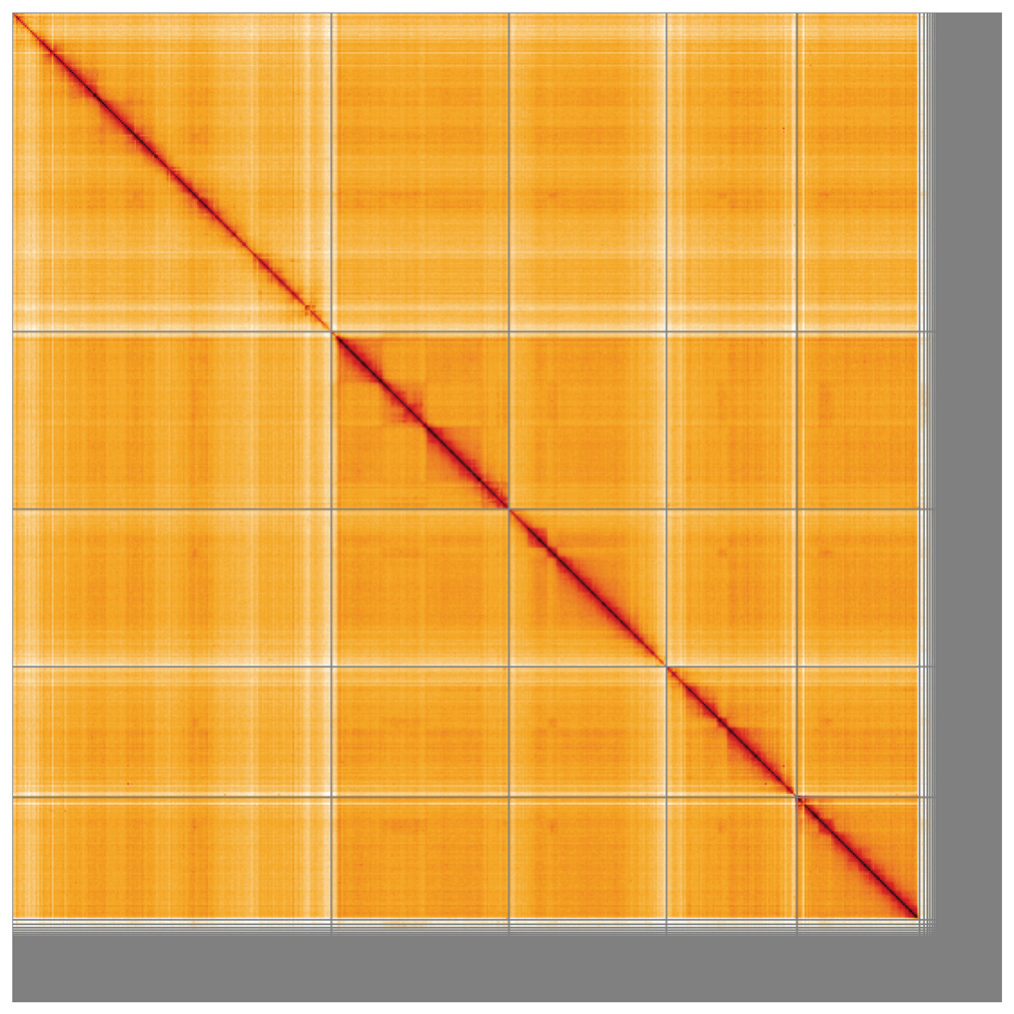
Genome assembly of *Dicyrtomina minuta* qeDicMinu4.1: Hi-C contact map of the qeDicMinu4.1 assembly, visualised using HiGlass. Chromosomes are shown in order of size from left to right and top to bottom. An interactive version of this figure may be viewed at https://genome-note-higlass.tol.sanger.ac.uk/l/?d=cRUc7wPZQ9e5NHpn5R-dYg.

The final assembly has a total length of 582.0 Mb in 485 sequence scaffolds with a scaffold N50 of 89.8 Mb ( Table 1). The snail plot in Figure 1 provides a summary of the assembly statistics, while the distribution of assembly scaffolds on GC proportion and coverage is shown in Figure 2. The cumulative assembly plot in Figure 3 shows curves for subsets of scaffolds assigned to different phyla. Most (88.88%) of the assembly sequence was assigned to 5 chromosomal-level scaffolds, representing three autosomes and the X _1_ and X _2_ sex chromosomes. Chromosome-scale scaffolds confirmed by the Hi-C data are named in order of size ( Figure 4; Table 2). Chromosomes X _1_ and X _2_ were identified based on synteny with *Allacma fusca* (GCA_947179485.1) ( Jaron *et al.*, 2023). While not fully phased, the assembly deposited is of one haplotype. Contigs corresponding to the second haplotype have also been deposited. The mitochondrial genome was also assembled and can be found as a contig within the multifasta file of the genome submission.

**Table 1.** Genome data for *Dicyrtomina minuta*, qeDicMinu4.1.

Project accession data		
Assembly identifier	qeDicMinu4.1	
Species	*Dicyrtomina minuta*	
Specimen	qeDicMinu4	
NCBI taxonomy ID	1387116	
BioProject	PRJEB58248	
BioSample ID	Genome sequencing: SAMEA7701774 Hi-C scaffolding: SAMEA7701781	
Isolate information	qeDicMinu4: whole organism (genome sequence)	
Assembly metrics *		*Benchmark*
Consensus quality (QV)	60.5	*≥ 50*
*k*-mer completeness	100.0%	*≥ 95%*
BUSCO **	C:95.1%[S:91.8%,D:3.3%],F:2.0%,M:2.9%,n:1013	*C ≥ 95%*
Percentage of assembly mapped to chromosomes	88.88%	*≥ 95%*
Sex chromosomes	X _1_, X _2_	*localised homologous pairs*
Organelles	Mitochondrial genome: 15.59 kb	*complete single alleles*
Raw data accessions		
PacificBiosciences Sequel IIe	ERR10879914	
Hi-C Illumina	ERR10684080	
Genome assembly		
Assembly accession	GCA_949802685.1	
*Accession of alternate haplotype*	GCA_951387605.1	
Span (Mb)	582.0	
Number of contigs	1325	
Contig N50 length (Mb)	1.0	
Number of scaffolds	485	
Scaffold N50 length (Mb)	89.8	
Longest scaffold (Mb)	181.99	

* Assembly metric benchmarks are adapted from column VGP-2020 of “Table 1: Proposed standards and metrics for defining genome assembly quality” from Rhie *et al.* (2021). ** BUSCO scores based on the arthropoda_odb10 BUSCO set using version 5.3.2. C = complete [S = single copy, D = duplicated], F = fragmented, M = missing, n = number of orthologues in comparison. A full set of BUSCO scores is available at https://blobtoolkit.genomehubs.org/view/GCA_949802685.1/dataset/qeDicMinu4_1/busco.

**Table 2.** Chromosomal pseudomolecules in the genome assembly of *Dicyrtomina minuta*, qeDicMinu4.

INSDC accession	Name	Length (Mb)	GC%
OX461808.1	1	181.99	35.0
OX461810.1	2	89.75	35.5
OX461811.1	3	74.52	35.5
OX461813.1	MT	0.02	27.5
OX461809.1	X1	101.2	36.0
OX461812.1	X2	69.85	36.5

The estimated Quality Value (QV) of the final assembly is 60.5 with *k*-mer completeness of 100.0%, and the assembly has a BUSCO v5.3.2 completeness of 95.1% (single = 91.8%, duplicated = 3.3%), using the arthropoda_odb10 reference set ( *n* = 1,013).

Metadata for specimens, barcode results, spectra estimates, sequencing runs, contaminants and pre-curation assembly statistics are given at https://links.tol.sanger.ac.uk/species/1387116.

## Methods

### Sample acquisition and nucleic acid extraction

Specimens of *Dicyrtomina minuta* were collected from Wytham Woods, Oxfordshire, UK (latitude 51.78, longitude –1.34) on 2020-08-03 by aspiration. The specimens were collected and identified by Kamil Jaron (Wellcome Sanger Institute) and preserved on dry ice. The specimen used for genome sequencing was a female adult *Dicyrtomina minuta* (specimen ID Ox000721, ToLID qeDicMinu4), and a second specimen (ID Ox000728, ToLID qeDicMinu3) was used for Hi-C sequencing.

The workflow for high molecular weight (HMW) DNA extraction at the Wellcome Sanger Institute (WSI) Tree of Life Core Laboratory includes a sequence of core procedures: sample preparation; sample homogenisation, DNA extraction, fragmentation, and clean-up. In sample preparation, the qeDicMinu4 sample was weighed and dissected on dry ice ( Jay *et al.*, 2023). Tissue from the whole organism was homogenised using a PowerMasher II tissue disruptor ( Denton *et al.*, 2023a). HMW DNA was extracted using the Automated MagAttract v1 protocol ( Sheerin *et al.*, 2023). DNA was sheared into an average fragment size of 12–20 kb in a Megaruptor 3 system with speed setting 30 ( Todorovic *et al.*, 2023). Sheared DNA was purified by solid-phase reversible immobilisation ( Strickland *et al.*, 2023): in brief, the method employs AMPure PB beads to eliminate shorter fragments and concentrate the DNA. The concentration of the sheared and purified DNA was assessed using a Nanodrop spectrophotometer and Qubit Fluorometer and Qubit dsDNA High Sensitivity Assay kit. Fragment size distribution was evaluated by running the sample on the FemtoPulse system.

Protocols developed by the WSI Tree of Life laboratory are publicly available on protocols.io ( Denton *et al.*, 2023b).

### Sequencing

Pacific Biosciences HiFi circular consensus DNA sequencing libraries were constructed according to the manufacturers’ instructions. DNA sequencing was performed by the Scientific Operations core at the WSI on a Pacific Biosciences Sequel IIe instrument. Hi-C data were also generated from whole organism tissue of qeDicMinu3 using the Arima v2 kit. The Hi-C sequencing was performed using paired-end sequencing with a read length of 150 bp on the Illumina NovaSeq 6000 instrument.

### Genome assembly and curation

Assembly was carried out with Hifiasm ( Cheng *et al.*, 2021) and haplotypic duplication was identified and removed with purge_dups, without the -e option ( Guan *et al.*, 2020). The assembly was then scaffolded with Hi-C data ( Rao *et al.*, 2014) using YaHS ( Zhou *et al.*, 2023). The assembly was checked for contamination and corrected as described previously ( Howe *et al.*, 2021). Manual curation was performed using HiGlass ( Kerpedjiev *et al.*, 2018) and PretextView ( Harry, 2022). The mitochondrial genome was assembled using MitoHiFi ( Uliano-Silva *et al.*, 2023), which runs MitoFinder ( Allio *et al.*, 2020) or MITOS ( Bernt *et al.*, 2013) and uses these annotations to select the final mitochondrial contig and to ensure the general quality of the sequence.

### Evaluation of final assembly

A Hi-C map for the final assembly was produced using bwa-mem2 ( Vasimuddin *et al.*, 2019) in the Cooler file format ( Abdennur & Mirny, 2020). To assess the assembly metrics, the *k*-mer completeness and QVy consensus quality values were calculated in Merqury ( Rhie *et al.*, 2020). This work was done using Nextflow ( Di Tommaso *et al.*, 2017) DSL2 pipelines “sanger-tol/readmapping” ( Surana *et al.*, 2023a) and “sanger-tol/genomenote” ( Surana *et al.*, 2023b). The genome was analysed within the BlobToolKit environment ( Challis *et al.*, 2020) and BUSCO scores ( Manni *et al.*, 2021; Simão *et al.*, 2015) were calculated.

Table 3 contains a list of relevant software tool versions and sources.

**Table 3.** Software tools: versions and sources.

Software tool	Version	Source
BlobToolKit	4.2.1	https://github.com/blobtoolkit/blobtoolkit
BUSCO	5.3.2	https://gitlab.com/ezlab/busco
Hifiasm	0.16.1-r375	https://github.com/chhylp123/hifiasm
HiGlass	1.11.6	https://github.com/higlass/higlass
Merqury	MerquryFK	https://github.com/thegenemyers/MERQURY.FK
MitoHiFi	2	https://github.com/marcelauliano/MitoHiFi
PretextView	0.2	https://github.com/wtsi-hpag/PretextView
purge_dups	1.2.3	https://github.com/dfguan/purge_dups
sanger-tol/genomenote	v1.0	https://github.com/sanger-tol/genomenote
sanger-tol/readmapping	1.1.0	https://github.com/sanger-tol/readmapping/tree/1.1.0
YaHS	1.2a	https://github.com/c-zhou/yahs

### Wellcome Sanger Institute – Legal and Governance

The materials that have contributed to this genome note have been supplied by a Darwin Tree of Life Partner. The submission of materials by a Darwin Tree of Life Partner is subject to the **‘Darwin Tree of Life Project Sampling Code of Practice’**, which can be found in full on the Darwin Tree of Life website here. By agreeing with and signing up to the Sampling Code of Practice, the Darwin Tree of Life Partner agrees they will meet the legal and ethical requirements and standards set out within this document in respect of all samples acquired for, and supplied to, the Darwin Tree of Life Project. 

Further, the Wellcome Sanger Institute employs a process whereby due diligence is carried out proportionate to the nature of the materials themselves, and the circumstances under which they have been/are to be collected and provided for use. The purpose of this is to address and mitigate any potential legal and/or ethical implications of receipt and use of the materials as part of the research project, and to ensure that in doing so we align with best practice wherever possible. The overarching areas of consideration are:

• Ethical review of provenance and sourcing of the material

• Legality of collection, transfer and use (national and international) 

Each transfer of samples is further undertaken according to a Research Collaboration Agreement or Material Transfer Agreement entered into by the Darwin Tree of Life Partner, Genome Research Limited (operating as the Wellcome Sanger Institute), and in some circumstances other Darwin Tree of Life collaborators.

## Data Availability

European Nucleotide Archive: *Dicyrtomina minuta*. Accession number PRJEB58248; https://identifiers.org/ena.embl/PRJEB58248 ( Wellcome Sanger Institute, 2023). The genome sequence is released openly for reuse. The *Dicyrtomina minuta* genome sequencing initiative is part of the Darwin Tree of Life (DToL) project. All raw sequence data and the assembly have been deposited in INSDC databases. The genome will be annotated using available RNA-Seq data and presented through the Ensembl pipeline at the European Bioinformatics Institute. Raw data and assembly accession identifiers are reported in Table 1.

## Author information

Members of the University of Oxford and Wytham Woods Genome Acquisition Lab are listed here: https://doi.org/10.5281/zenodo.7125292.

Members of the Darwin Tree of Life Barcoding collective are listed here: https://doi.org/10.5281/zenodo.4893703.

Members of the Wellcome Sanger Institute Tree of Life Management, Samples and Laboratory team are listed here: https://doi.org/10.5281/zenodo.10066175.

Members of Wellcome Sanger Institute Scientific Operations: Sequencing Operations are listed here: https://doi.org/10.5281/zenodo.10043364.

Members of the Wellcome Sanger Institute Tree of Life Core Informatics team are listed here: https://doi.org/10.5281/zenodo.10066637.

Members of the Tree of Life Core Informatics collective are listed here: https://doi.org/10.5281/zenodo.5013541.

Members of the Darwin Tree of Life Consortium are listed here: https://doi.org/10.5281/zenodo.4783558.
