# Supplementary material for: The genome sequence of the springtail, Dicyrtomina minuta (O.Fabricius, 1783)
Source: Wellcome Open Res. 2024 Jul 29;9:417. [Version 1] doi: 10.12688/wellcomeopenres.22765.1 (PMC11411247; doi:10.12688/wellcomeopenres.22765.1)
Supplement: Peer review history

# Reviewer report: Reviewer response for version 1

**DOI:** 10.21956/wellcomeopenres.25069.r97699

**Published:** 2024-09-18

**Authors:** José M Martín-Durán

**Affiliations:** 1 Queen Mary University of London, London, UK

This Genome Note reports the sequencing and assembly of the genome of the springtail (Collembola) *Dicyrtomina minuta. *Collembolans are fascinating animals that occupy a key phylogenetic position to investigate the evolution of Insecta, the most diverse animal clade on Earth.

 Strengths

 - High-quality genome for an important and understudied animal group.

 - Important resource to investigate sex determination and karyotype evolution in Collembola.

 Weaknesses

 - None for this resource. The fact that the karyotype inferred from genome sequence differs so dramatically from those observed in other species warrants further investigations and experimental validations.

Are sufficient details of methods and materials provided to allow replication by others?

Yes

Is the rationale for creating the dataset(s) clearly described?

Yes

Are the datasets clearly presented in a useable and accessible format?

Yes

Are the protocols appropriate and is the work technically sound?

Yes

Reviewer Expertise:

Comparative genomics, evolutionary developmental biology, invertebrates

I confirm that I have read this submission and believe that I have an appropriate level of expertise to confirm that it is of an acceptable scientific standard.

# Reviewer report: Reviewer response for version 1

**DOI:** 10.21956/wellcomeopenres.25069.r95172

**Published:** 2024-09-01

**Authors:** Alex Makunin

**Affiliations:** 1 Wellcome Sanger Institute, Hinxton, England, UK

The data note by Jaron et al. presents a high quality chromosome scale assembly for Collembola species Dicyrtomina minuta. This is the first chromosomal genome assembly for Dicyrtomidae family. The presentation of the results and methods is standardized and overall very transparent. 

**I have a few minor suggestions:**

- Is it possible to specify the amount of DNA used for PacBio sequencing? From the methods section it seems that the no amplification was done prior to sequencing, i.e. low input protocol was used.
- The identification of X1 and X2 chromosomes is based on genome alignment to Allacma fusca, also from DToL project (Jaron et al., 2023). However, in that publication the source of chromosome identities is not specified, so it seems reasonable to include more information on sex chromosomes delineation - I assume it was based on coverage given that a male Allacma fusca was used for assembly.
- It might be worth including a synteny plot as a figure, possibly accompanied by information on expected divergence times between Dicyrtomina (Dicyrtomidae) and Allacma (Sminthuridae), e.g. estimate of ca 280MY from mitochondrial genomes (Leo et al., 2019).
- It might be interesting to include a short discussion around expected chromosome numbers and their evolutionary variability. For example, D. ornata females were reported to have 2n = 12 (Dallai et al 1999) - one chromosome more than reported for D. minuta here, although in introduction it is mentioned that both were considered as a single species at some point

Are sufficient details of methods and materials provided to allow replication by others?

Partly

Is the rationale for creating the dataset(s) clearly described?

Yes

Are the datasets clearly presented in a useable and accessible format?

Yes

Are the protocols appropriate and is the work technically sound?

Yes

Reviewer Expertise:

Comparative genomics

I confirm that I have read this submission and believe that I have an appropriate level of expertise to confirm that it is of an acceptable scientific standard.

## References

[1] : Mitochondrial Genome Diversity in Collembola: Phylogeny, Dating and Gene Order. *Diversity* .2019;11(9) : 10.3390/d11090169 10.3390/d11090169

[2] : Chromosome elimination and sex determination in springtails (Insecta, Collembola). *J Exp Zool* .1999;285(3) : 10.1002/(sici)1097-010x(19991015)285:3<215::aid-jez4>3.0.co;2-5 215-25 10.1002/(sici)1097-010x(19991015)285:3<215::aid-jez4>3.0.co;2-5 10497320

[3] : The genome sequence of the springtail Allacma fusca (Linnaeus, 1758). *Wellcome Open Res* .2023;8: 10.12688/wellcomeopenres.19690.1 319 10.12688/wellcomeopenres.19690.1 37593568 PMC10427803
